# Eosinophilic infiltrate in a patient with severe *Legionella *pneumonia as a levofloxacin-related complication: a case report

**DOI:** 10.1186/1752-1947-4-360

**Published:** 2010-11-11

**Authors:** Nicola Facciolongo, Francesco Menzella, Claudia Castagnetti, Alberto Cavazza, Roberto Piro, Cristiano Carbonelli, Luigi Zucchi

**Affiliations:** 1 Department of Pneumology, S. Maria Nuova Hospital, 42123 Reggio Emilia, Italy; 2 Department of Pathology, S. Maria Nuova Hospital, 42123 Reggio Emilia, Italy

## Abstract

**Introduction:**

*Legionella *pneumonia can appear with different levels of severity and it can often present with complications such as acute respiratory distress syndrome.

**Case presentation:**

We report the case of a 44-year-old Caucasian man with *Legionella *pneumonia with successive development of severe acute respiratory distress syndrome. During his stay in intensive care the clinical and radiological situation of the previously observed acute respiratory distress syndrome unexpectedly worsened due to acute pulmonary eosinophilic infiltrate of iatrogenic origin.

**Conclusion:**

Levofloxacin treatment caused the occurrence of acute eosinophilic infiltrate. Diagnosis was possible following bronchoscopic examination using bronchoaspirate and transbronchial biopsy.

## Introduction

Since the pneumonia epidemic that struck the delegates of the American Legion Convention in Philadelphia in 1976, *Legionella *spp. has become a relatively frequent cause of community acquired pneumonia [1].

*Legionella *may appear in different forms, from subclinical presentations to Legionnaires' disease, which has a mortality rate as high as 30 to 50% in cases of hospital infections and in cases of complications such as acute respiratory distress syndrome (ARDS). The fatality rate is 5 to 25% even in patients who are immunocompetent [2].

Other complications are rare, although a significant number of drugs used in the treatment of *Legionella *pneumonia can be associated with the appearance of pulmonary eosinophilic infiltrates, especially non-steroidal anti-inflammatory drugs (NSAIDs) and antibiotics [3]. The diagnosis is mainly based on the temporal correlation between the administration of drugs and the appearance of the clinical condition, but it is often not easy to determine the etiologic agent with certainty.

This report concerns the case of a man with *Legionella *pneumonia that evolved into ARDS and then became complicated with eosinophilic infiltration as an effect of treatment with levofloxacin. Usually this drug is safe, though in some cases can cause eosinophilic pneumonia [4].

## Case presentation

A 44-year-old Caucasian man presented to our hospital for hyperpyrexia (over 39°C) for about a week, with general weakness and strong headaches; he had been treated by his general practitioner with amoxicillin and clavulanate administrated orally with no improvement.

His case history revealed that he was a smoker (20 packs/year). No other pathologies or trips abroad had been registered in the last 6 months.

On admission, he had hyperpyrexia (38.9°C), headache, dry cough, diarrhea, general weakness and sinus tachycardia (100 beats/minute); his oxygen saturation was 95% (no oxygen supplement).

**Figure 1:**
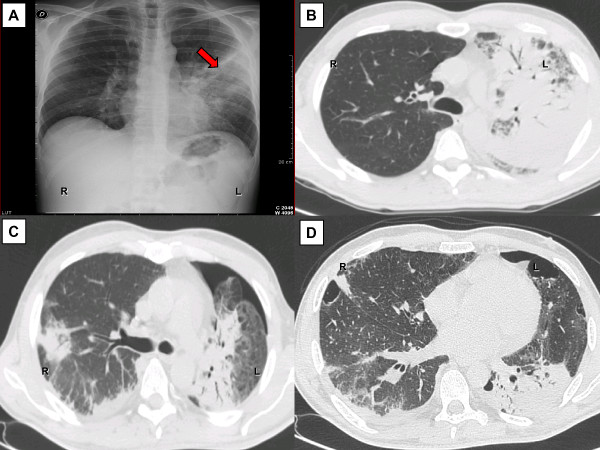
**Chest X-ray and computed tomography (CT) images**. A) A chest X-ray taken on admission: extensive pulmonary consolidation can be seen in the upper left lobe (arrow). There was an absence of pleural effusion and no cardiomegaly. B) A chest CT scan taken on the ninth day: consolidation areas can be seen on the whole superior left lobe, mixed with ground-glass areas and air bronchogram. There was an absence of pleural effusion. C, D) A CT scan taken on the 21st day: on the left there is parenchymal consolidation with air bronchogram and pneumothorax, and several areas of parenchymal consolidation on the right superior lobe. There was an absence of pleural effusion.

The results of a physical examination of his chest were reduced vesicular respiration and crackling in the median axillary line to the left and in front; a chest X-ray showed extensive inconsistent parenchymal consolidation at the fissure of the left upper lobe (Figure 1A).

The results of initial laboratory examinations revealed his white blood cell count was 2020 cells/mm^3^, total bilirubin level was (1.6 mg/dL), he had reduced albuminemia (2.7 g/dL), increased alkaline phosphatase (382 U/L), γ-glutamyl transferase (69 U/L) and creatine phosphokinase (422 U/L). His serology test results were negative for Hepatitis B virus, Hepatitis C virus and HIV. His initial blood culture test results were negative for aerobic and anaerobic germs and mycetes.

Our patient began treatment with intravenous piperacillin and tazobactam (13.5 g/day) and clarithromycin orally (1 g/day). On the third day the results of his urinary antigen test were found to be positive for *Legionella *serogroup 1, so clarithromycin was suspended and substituted with intravenous levofloxacin (750 mg/day). We maintained the piperacillin and tazobactam treatment to help prevent secondary infection from other Gram-positive and Gram-negative bacteria.

On the sixth day, his clinical condition worsened. After consultation with an infectious disease specialist, we added rifampicin (900 mg/day) to support the levofloxacin action against *Legionella *pneumonia.

On the ninth day he showed respiratory distress (40 breaths/minute). An Arterial Blood Gas analysis in room air gave the following results: partial O_2 _pressure (pO_2_) of 50 mmHg, partial CO_2 _pressure (pCO_2_) of 30 mmHg, pH 7.50 and oxygen saturation (SaO_2_) of 86%.

A computed tomography (CT) scan of his chest revealed multiple areas of parenchymal consolidation in the entire upper left pulmonary lobe, mixed with ground-glass areas and abundant pleural effusion. In the right lung, in the dorsal and basal regions, there were ground-glass areas mixed with consolidation areas (Figure 1B).

On the 10th day PaO_2_/fraction of inspired O_2 _(FiO_2_) ratio was 101 and he was moved to our intensive care unit. Here he was placed on a ventilator on continuous positive airway pressure modality, with noticeable improvement of the respiratory parameters (PaO_2_/FiO_2 _ratio of 254). On the 17th day, levofloxacin was suspended in order to allow wash-out and taking of further blood cultures. On the 19th day levofloxacin was resumed; after advice from an infectious diseases specialist intravenous levofloxacin 1500 mg per day together with intravenous fluconazole 800 mg per day were given

On the 21st day, after an initial improvement, he showed respiratory distress. A CT scan showed increased parenchymal consolidation with left pneumothorax (Figure 1C, D).

On the 22nd day, because of the unexpected occurrence of muscular exhaustion, orotracheal intubation was performed and he was placed on a mechanical ventilator in synchronized intermittent mandatory ventilation mode associated with appropriate kinetic therapy on a reclining bed. A fibrobronchoscopy study, carried out with bronchoalveolar lavage (BAL) for bacteriological reasons and in order to define the cytological profile, revealed the presence of numerous macrophages (32%), lymphocytes (26%; CD4/CD8 ratio 0.8), neutrophilic granulocytes (40%) and some eosinophilic granulocytes (2%). Protozoa, fungus and neoplastic cells were absent.

On the 23rd day, methylprednisolone (120 mg/day intravenously) was added to the therapy.

**Figure 2:**
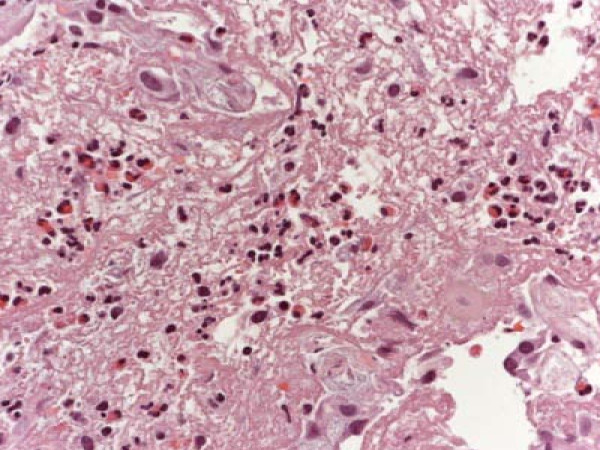
**Histological images**. Transbronchial biopsies showed several eosinophils associated with fibrin (hematoxylin and eosin stain, 200×).

**Figure 3:**
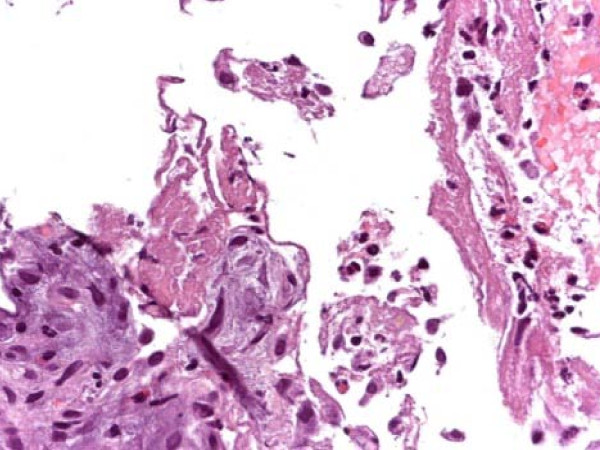
**Histological images**. Focally, hyaline membranes were present (hematoxylin and eosin stain, 200×).

On the 26th day, he underwent another bronchoscopy, with BAL and transbronchial biopsy in the basal segments of the lower right lobe, which revealed a histological condition compatible with acute eosinophilic pneumonia (Figures 2 and 3). The BAL confirmed the presence of eosinophils 28%, macrophages 57%, lymphocytes 15%, neutrophilic granulocytes 2% and a CD4/CD8 ratio of 1. Incidental findings showed masses of finely pigmented macrophages (due to our patient's smoking habit). Serum levels of total IgE were within normal limits, and the specific IgE antibody results for allergens (food, pollen, fungal) were also negative. Fecal and serological test results were negative for parasites.

On the 27th day, his steroid therapy was increased (methylprednisolone 1 g/day) while levofloxacin was suspended. His response to steroid therapy was rapid, with a general improvement starting from the fifth day of treatment (the 32nd day overall), associated with accompanying improvement of respiratory exchange and subsequent return to spontaneous breathing on the 41st day (PaO_2_/FiO_2 _ratio of 357).

**Figure 4:**
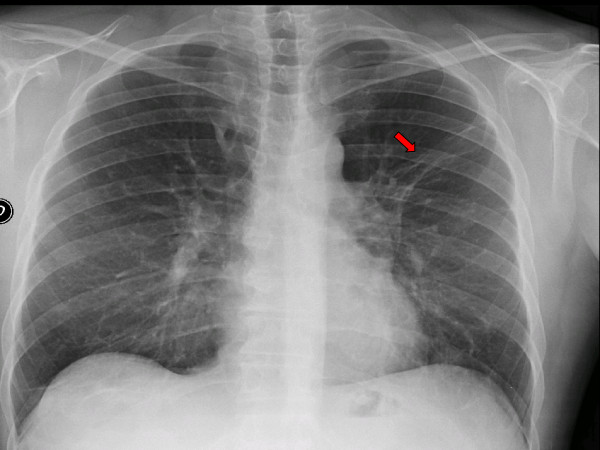
**Chest X-ray**. Thickening areas and parenchymal distortion can be seen on the left upper lobe. Diffuse thickening can be seen on medial and lower lobes (arrow).

On the 51st day, a chest X-ray showed that the pneumonia bilateral consolidation had completely resolved (Figure 4).

## Discussion

ARDS is a common medical emergency and is usually a complication of a previous illness, which is the etiological cause [5]. In our patient, the unusual fact was the overlapping of acute eosinophilic infiltrate in legionellosis.

Eosinophilic pneumonias include a wide range of pulmonary pathologies, characterized by alveolar and peripheral blood eosinophilia. Peripheral eosinophilia may be absent, in particular in the early stages of acute idiopathic eosinophilia pneumonia or in patients taking systemic corticosteroids. It may occur with extremely variable forms of seriousness, from asymptomatic pulmonary infiltrates to acute respiratory distress syndrome associated with respiratory insufficiency. The possible causes, such as drugs or parasitic infections, have been widely studied, but are, in most cases, idiopathic [6]. In our opinion, in accordance with the findings of other authors [7], early low-dose steroid therapy leads to a better outcome of pneumonia with severe respiratory distress; however it could determine a delayed onset of eosinophilic pneumonia.

In our patient, we are inclined to consider it as having an iatrogenic etiopathogenesis. Other causes were excluded by laboratory tests for differential diagnosis options (serum total and specific IgE, fecal and serologic examinations for parasite infections).

Eosinophilic pneumonia has been linked to more than 80 drugs, although only 20 of these (for the most part NSAIDs and antibiotics) can be considered as common causes of this pathology [6]. All the drugs administered in the weeks prior to the appearance of eosinophilic infiltrate should be suspected as a possible cause of the pathology. Iatrogenic eosinophilic infiltrates usually develop progressively, with dyspnea, cough and fever in subjects who have taken certain drugs for weeks or months.

The diagnosis of drug-induced eosinophilic pneumonia is mainly based on a detailed history of drug exposure, evidence of eosinophil accumulation in the lung and exclusion of other causes. Numerous methods have been studied in order to demonstrate sensitivity to one or more drugs. One of the most commonly applied methods is the lymphocyte stimulation test (LST), which measures the proliferation of T lymphocytes in response to a drug *in vitro*, in order to diagnose a previous reaction *in vivo*. This concept was confirmed by the finding of drug-specific T lymphocyte clones that can interact with cellular receptors without being metabolized and without bonding to protein carriers [8].

We did not consider it necessary to carry out the LST with our patient because this method is not specific and sensitive, and it has the major drawback of being difficult to interpret [8].

With regard to the challenge test *in vivo*, this was not performed because of the serious clinical condition of our patient, who in any case did not give his consent. However, voluntary challenge may cause life-threatening adverse reactions and it should be limited to rare situations [9].

Among the possible causes we considered, the first was levofloxacin. There are some reports in the literature regarding the possibility of development of eosinophilic pneumonia during the course of levofloxacin therapy [4]; moreover, it was the drug administered to our patient for the greatest number of days (21 in total). Other points can be taken into account: (1) the drug was suspended for four days in order to allow for wash-out and subsequent blood culture; afterwards, the same drug was resumed. At the same time, the clinical radiological findings became worse, with an unintentional challenge effect. (2) The BAL on the 22nd day, as some other authors have reported, still showed compatibility with ARDS *Legionella*, [10] while the following BAL showed eosinophilia (28%) compatible with an acute eosinophilic pneumonia [6], which histological exams confirmed (Figure 3).

With regard to the other drugs administered, there are reports of isolated cases of eosinophilia associated with parenchymal infiltrates as a consequence of rifampicin therapy [11]. There is only one reported case where clarithromycin may have led to eosinophilic pneumonia [12], but our patient was only treated with this drug for two days. Moreover, it is possible that eosinophilic pneumonia could be an adverse reaction to smoking in predisposed subjects: this sometimes happens to patients who have recently started smoking or who have modified their 'way' of smoking (for example, increasing or changing type of smoking). Our patient, however, did not report any changes, either in quantity or in quality, in his smoking habits, so this would seem to exclude any relation to smoking [13].

However, it is plausible that smoking could have acted as a cofactor (together with the drugs) in triggering the clinical condition, because it is a known fact that acute eosinophilic infiltrates are often frequent in smokers [14].

## Conclusion

In conclusion, levofloxacin may be the most probable cause of the occurrence of acute eosinophilic infiltrate in this patient. It is important to emphasize that we decided to change the diagnostic and therapeutic approach only when the presence of eosinophilic infiltrate was proven by transbronchial biopsy. Published studies dealing with risks of invasive endoscopic procedures in a patient who was critically ill on mechanical ventilation showed a higher incidence of complications such as hemorrhage and pneumothorax. Correlating the endoscopic risk to the percentage of correctly carried out diagnoses, which varies from 33% to 76%, with consequent change in therapeutic strategy, it may be stated that the risk/benefit ratio of the endoscopic procedure in terms of therapeutic response is surely in its favor and it is, therefore, recommended [15].

## Competing interests

The authors declare that they have no competing interests.

## Authors' contributions

NF coordinated diagnostic and therapeutic stages and was one of the principal contributors in writing the manuscript. MF contributed to the clinical approach, analyzed and interpreted the data and was a major contributor in writing the manuscript. CC was a contributor in writing the manuscript. AC performed the histological examination of the lung and was a contributor in writing the manuscript. CC was a contributor in writing the manuscript. RP was a contributor in writing the manuscript. LZ was a contributor in writing the manuscript and he gave final approval of the version to be published. All authors read and approved the final manuscript.

## Consent

Written informed consent was obtained from the patient for publication of this case report and any accompanying images. A copy of the written consent is available for review by the Editor-in-Chief of this journal.
